# Study of Oxidant/Antioxidant Profile in Dogs with Mammary Cancer Undergoing Mastectomy, During the Peri-Operative Period †

**DOI:** 10.3390/vetsci12060562

**Published:** 2025-06-08

**Authors:** Angelos-Lauris Thomas, Maria Karayannopoulou, Tilemachos Anagnostou, Dimitra Psalla, Konstantinos Ioannou, Argyrios Ginoudis, Ioannis Savvas, Dimitra Pardali

**Affiliations:** 1 Department of Clinical Studies-Companion Animal Clinic, School of Veterinary Medicine, Faculty of Health Sciences, Aristotle University of Thessaloniki, 11 St. Voutyra Street, 546 27 Thessaloniki, Greece; marikara@vet.auth.gr (M.K.); tanagnos@vet.auth.gr (T.A.); kon_ioan@yahoo.gr (K.I.); isavas@vet.auth.gr (I.S.); 2 Laboratory of Pathology, School of Veterinary Medicine, Faculty of Health Sciences, Aristotle University of Thessaloniki, University Campus, 541 24 Thessaloniki, Greece; dpsalla@vet.auth.gr; 3 Department of Clinical Studies-Diagnostic Laboratory, School of Veterinary Medicine, Faculty of Health Sciences, Aristotle University of Thessaloniki, 11 St. Voutyra Street, 546 27 Thessaloniki, Greece; ginoudisa@gmail.com (A.G.); dpardali@vet.auth.gr (D.P.)

**Keywords:** canine mammary cancer, mastectomy, Reactive Oxygen Species (ROS), oxidative stress, blood oxidant/antioxidant profile, peri-operative period, micro-metastases

## Abstract

In cancer cases, any imbalance between Reactive Oxygen Species (ROS) production and host antioxidant capacity (oxidative stress), during the immediate post-operative period, is involved in cancer cell dissemination (micro-metastases). Surgical trauma is considered the main cause for ROS production during that period. Increased oxidative stress has been found in dogs with mammary neoplasms. Our objective was to investigate the blood oxidant/antioxidant profile in bitches with mammary cancer, during the peri-operative period, hypothesizing that oxidative stress would be further increased after mastectomy. Twelve bitches with mammary cancer and no distant metastases, with a comparable extent of surgical wound and receiving the same anesthetic protocol, were included. Their serum oxidant/antioxidant profile was evaluated chromatometrically using the d-ROMs (Reactive Oxygen Metabolite-derived compounds) and the BAP (biological antioxidant potential) tests, on the 1st day (before/after surgery), and on the 2nd, 3rd and 10th days post-operatively. The ratio of d-ROMs and BAP values (oxidative stress index; OSI) was also calculated. Statistically significant differences (increases) were found only in d-ROMS on the second and third post-operative day compared to the first day (before surgery) of measurement (*p* = 0.007 and *p* = 0.002, respectively). Our findings indicate a post-surgical overproduction of ROS and therefore an oxidative stress increase potentially affecting patient outcome.

## Simple Summary

In cancer cases, any imbalance between Reactive Oxygen Species (ROS) production and host antioxidant capacity (oxidative stress), during the immediate post-operative period, is involved in cancer cell dissemination (micro-metastases); surgical trauma is considered the main cause for ROS production. Our objective was to investigate the blood oxidant/antioxidant profile in bitches with mammary cancer, hypothesizing that oxidative stress would be increased after mastectomy. In twelve bitches with mammary cancer, serum oxidant/antioxidant profile was evaluated chromatometrically using the d-ROMs (Reactive Oxygen Metabolite-derived compounds) and the BAP (biological antioxidant potential) tests, on the 1st day (before/after surgery), and on the 2nd, 3rd and 10th days post-operatively. Statistically significant differences (increases) were found in d-ROMS on the second and third post-operative day compared to the first day (before surgery) of measurement (*p* = 0.007 and *p* = 0.002, respectively). Our findings indicate a post-surgical oxidative stress increase potentially affecting patient outcome.

## 1. Introduction

Mastectomy is the only or the main treatment in most canine mammary cancer cases. However, it is well known from human and animal studies that surgery itself, as well as some drugs used in anesthesia/analgesia, induce immunosuppression during the immediate post-operative period potentially allowing residual cancer cells to proliferate and metastasize [1,2,3,4,5,6,7,8]. It has been reported that Reactive Oxygen Species (ROS) regulate many of the pathways which facilitate a number of cellular processes (adhesion, invasion, angiogenesis and proliferation) that are necessary for the successful dissemination of cancer cells during that period [9,10,11,12]. Surgical trauma is considered the main cause for ROS production [9,10,11,13]. Moreover, some drugs used in anesthesia for mastectomy can also induce ROS production [14,15].

Antioxidants from endogenous or exogenous origin act as a protective mechanism against the accumulation of ROS and the damage that they may induce, and contribute to the host immune defense. Any imbalance between ROS production and host antioxidant capacity that leads to the accumulation of ROS is defined as oxidative stress and is implicated in tumor growth and progression of the disease [10,11,13,16,17,18].

Tumor cells produce elevated levels of ROS compared to normal cells [11,19,20]. It has also been reported that in cancer cell lines, an inefficiency of the intracellular antioxidant system was found due to mutations of antioxidant enzymes, facilitating elevated ROS production by cancer cells [10]. Increased oxidative stress has been reported in dogs with mammary neoplasms compared to healthy controls [21,22,23,24], as in women with breast cancer [17,19,25,26,27,28,29]. Consequently, the use of antioxidants, not only for cancer prevention but also in cancer treatment, has been evaluated in many studies [18,20,21,28,30,31].

The aim of this study was to investigate the oxidant/antioxidant status in the blood of dogs with mammary cancer subjected to mastectomy, during the peri-operative period. We hypothesized that the existing oxidative stress in canine mammary cancer cases would be further increased after mastectomy due to surgical trauma and anesthesia potentially facilitating tumor metastases.

## 2. Materials and Methods

### 2.1. Animals

The animals included in the present study were selected from bitches with mammary tumors admitted to the University Companion Animal Clinic for diagnosis and therapeutic management (mastectomy). Inclusion criteria were as follows: histologically diagnosed malignant tumor(s) evaluated post-surgically, absence of distant metastases (clinical stage I, II or III of the disease according to the World Health Organization clinical staging system TNM) [32], comparable extent of surgical wound in all animals, and use of the same anesthetic protocol for mastectomy. Exclusion criteria included any other concurrent disease or treatment (at least six months before surgery) that is known to induce immunosuppression or increase oxidative stress. The type of surgery performed for tumor(s) excision was unilateral total mastectomy or bilateral regional mastectomy depending on the number of tumors and mammary glands involved [33]. In order to compare surgical wounds between dogs of different body size and weight, we used the term “extent of surgical wound”. It was defined as the % ratio of the wound surface area in m^2^/the body surface area (BSA) in m^2^, where wound surface area = maximum wound length × maximum wound width and BSA = K × W^2/3^ × 10^−4^ (K: 10.1 for dogs; W: body weight in grams) [34]. An initial number of 16 bitches with mammary tumors were subjected to mastectomy and blood sampling; however, four bitches were excluded after the post-surgical histological diagnosis of benign tumors. Therefore, a total of twelve bitches with mammary cancer were finally included in this study.

The study protocol was approved by our University Research Committee according to international guidelines on animal ethics and welfare (Date: 28 September 2022/No: 244751/2022). In addition, the owner’s consent was obtained for each dog that participated in the study.

### 2.2. Anesthetic Protocol

In this study, the anesthetic protocol for mastectomy was a protocol commonly used in veterinary practice. More specifically, pre-anesthetic medication included acepromazine (Acepromazine; Alfasan, The Netherlands) 0.05 mg kg^−1^ given intramuscularly (IM), morphine (morphine sulfate; Famar SA, Thiva, Greece) 0.2 mg kg^−1^ IM and meloxicam (Metacam; Boehringer Ingelheim Vetmedica GmbH, Ingelheim, Germany) 0.1 mg kg^−1^ subcutaneously (SC). Lactated Ringer’s solution (L-R Lactated Ringer’s Injection; Vioser, Trikala, Greece) was administered intravenously (IV) via the cephalic vein at a rate of 5 mL kg^−1^ h^−1^ until the end of surgery and anesthesia. Moreover, cefazolin (Vifazolin; Vianex, Kifisia, Greece) 20 mg kg^−1^ was given IV as prophylactic antibiotic treatment. Induction of anesthesia was carried out with propofol (Propofol MCT/LCT Fresenius; Fresenius KabiHellas, Marousi, Greece) IV, at an initial dose of 2 mg kg^−1^ and additional doses of 1 mg kg^−1^ as needed, whereas maintenance was performed with administration of isoflurane (AErrane; Baxter Healthcare Ltd., Norfolk, UK) in 100% oxygen through an appropriate cuffed endotracheal tube that was connected to the appropriate breathing system of an anesthetic machine. For intra-operative analgesia, fentanyl (Fentanyl; Janssen Pharmaceutica NV, Beerse, Belgium) was given IV at 0.1 μg kg^−1^ min^−1^ via a constant rate infusion until the end of surgery. For post-operative pain relief, morphine 0.1 mg kg^−1^ was given IM every 4 h for one day and meloxicam 0.1 mg kg^−1^ SC or per os every 24 h for five days. Additional rescue analgesia (morphine) was administered if indicated by pain assessments (Short Form of the Glasgow Composite Measure Pain Scale) performed every 2 h for the first 48 h postoperatively. The duration of surgery and the duration of anesthesia were recorded in all animals.

### 2.3. Histological Evaluation

Representative specimens obtained from the excised tumor(s) and lymph nodes were routinely processed and stained with hematoxylin and eosin for histological examination. The tumors were classified according to the Misdorp and colleagues [35] validated system.

### 2.4. Blood Measurements

On the day of mastectomy (1st day) and after a 12 h over-night fasting, before the administration of pre-anesthetic medication and at the end of surgery (1st day/pre and 1st day/post), as well as on the 2nd, 3rd and 10th post-operative day, 6 mL of blood was collected from each animal via jugular venipuncture. One mL of that blood was collected in EDTA-containing tubes for measurement of absolute numbers of white blood cells (WBCs) and neutrophils (NEUT) in an automated hematology analyzer (ADVIA 120; Siemens, Germany). The remaining 5 mL of blood was collected in tubes without any anti-coagulant for obtaining serum. The serum was then stored at −80 °C until measurements for evaluation of the oxidant/antioxidant profile of each dog were performed.

The oxidant/antioxidant status of each dog, during the peri-operative period, was evaluated chromatometrically in a spectrophotometric analyzer (Free Carpe Diem, Diacron Labs S.r.I., Grosseto, Italy) by using the d-ROMs (Reactive Oxygen Metabolite-derived compounds) test and the BAP (biological antioxidant potential) test. The d-ROMs test (Diacron Labs S.r.I., Grosseto, Italy) is a sensitive and well-established test for measuring the oxidation generated by free radicals in a sample of blood serum (fresh/frozen) or plasma and is expressed in Carratelli units (1 CARR U = 0.08 mg hydrogen peroxide/dL) [36,37,38,39]. The BAP test (Diacron Labs S.r.I., Grosseto, Italy) carried out in fresh/frozen serum or plasma and expressed in μmol/L provides a quantitative evaluation of the host biological antioxidant potential attributable to substances of endogenous or exogenous origin (e.g., albumin, uric acid, bilirubin, glutathione, ascorbic acid, tocopherols, etc.) [37,39,40,41]. Another value that was calculated was the oxidative stress index (OSI), which is the ratio of d-ROMs and BAP values and is expressed in Arbitrary Units (AU). For OSI calculations, the BAP values had to be converted from μmol/L to mmol/L [39,42].

Before every run of d-ROMs and BAP measurements, two-level (high and low) control material provided by the manufacturer was used, as well as calibrator material in case the control was out of range. In order to assess assay repeatability, the coefficient of variation (CV) of the d-ROMs and BAP tests was calculated by measuring in triplicate each sample of the first three dogs included in the study.

### 2.5. Statistical Analysis

Apriori power calculation concerning d-ROMs values revealed that 12 dogs were enough for a power >80% to detect statistically significant differences. The Kolmogorov–Smirnov test was used for assessing the normality of data distribution. A General Linear Model for repeated measures was used for comparisons between measurement times for d-ROMs, as well as for WBCs and NEUT measurements. Regarding BAP measurements and OSI values, a nonparametric test (Related-Samples Friedman’s Two-Way Analysis of Variance by Ranks) was used. For all statistical analyses, commercially available software (SPSS version 19.0; IBM-SPSS Science, Chicago, IL, USA) was utilized and data were expressed as mean ± standard deviation (SD). Significant differences were declared at *p* ≤ 0.05.

## 3. Results

### 3.1. Animals: Clinical and Histological Characteristics of Mammary Tumors

Most of the bitches included in this study were mongrels (8/12). The animals were aged 8–14 years (mean ± SD: 10.67 ± 1.97) with a weight range from 7 to 28 Kg. Only five bitches were intact, whereas the remaining seven had undergone ovariohysterectomy 2–4 years before mastectomy, due to pyometra. According to TNM classification, four of the included animals had mammary cancer of clinical stage I, four of stage II and four of stage III. Unilateral total mastectomy was performed in nine cases and bilateral regional mastectomy in three cases. Histological examination revealed that 10 bitches had carcinomas, one had multiple malignant mixed tumors and one had a sarcoma tumor. Lymph node metastasis was found only in one carcinoma case.

The “extent of surgical wound” ranged from 4.20 to 6.57% (mean ± SD: 5.31 ± 0.8%) of BSA showing a comparable extent of surgical wound in all cases.

The duration of surgery was from 60 to 240 min (mean ± SD: 123.33 ± 53.5 min), and the duration of anesthesia was from 110 to 270 min (mean ± SD: 200.83 ± 58.2 min).

### 3.2. Blood Measurements

**Figure 1:**
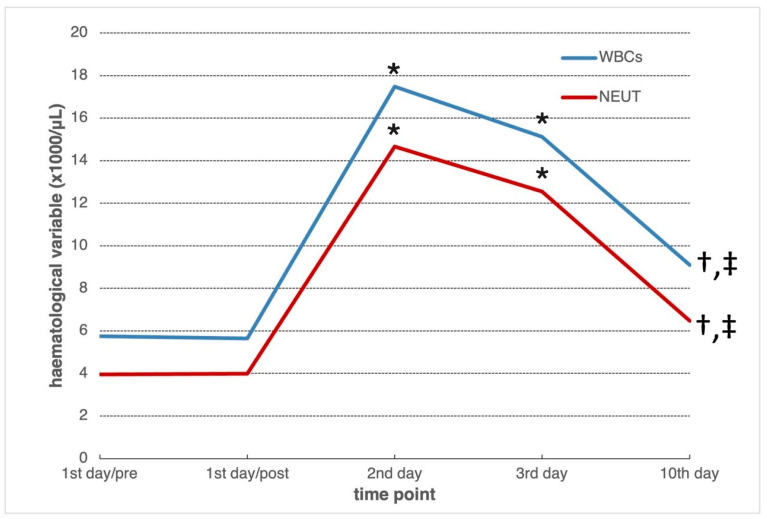
Absolute numbers (mean values × 10^3^/μL) of white blood cells (WBCs) and neutrophils (NEUT) in the blood of 12 bitches with mammary cancer undergoing mastectomy, measured prior to anesthesia (1st day/pre), at the end of surgery (1st day/post) and on 2nd, 3rd and 10th post-operative days. *: significant increase (*p* = 0.000) in WBCs and NEUT on 2nd and 3rd post-operative days compared to both 1st day values; †: significant decrease in WBCs (*p* = 0.011) and NEUT (*p* = 0.006) compared to 3rd post-operative day values; ‡: significant increase compared to 1st day values in WBCs (1st day/pre: *p* = 0.002 and 1st day/post: *p* = 0.005) and in NEUT (1st day/pre: *p* = 0.006 and 1st day/post: *p* = 0.014).

The mean absolute numbers of WBCs and NEUT at each measurement time are presented in Figure 1. There was a statistically significant increase (*p* = 0.000) in both variables on the second and third post-operative day compared to both first day values (pre- and post-surgery). A peak of these increases was observed on day 2. On the 10th post-operative day, the mean absolute number of WBCs, although this decreased significantly compared to day 3 (*p* = 0.011), was still significantly higher than the 1st day/pre (*p* = 0.002) and 1st day/post (*p* = 0.005) values. Similarly, on the same day (10th), the mean absolute number of NEUT was found to be decreased (*p* = 0.006) compared to day 3, but it was higher than the first day values (first day/pre: *p* = 0.006 and first day/post: *p* = 0.014).

The CV for d-ROMs and BAP tests was 3.2% and 5.1%, respectively. The results of d-ROMs measurements are presented in Table 1. Statistically significant differences (increased values) were found in d-ROMs on the second and third post-operative day compared to the first day/pre measurement (*p* = 0.007 and *p* = 0.002, respectively), as well as between the third post-operative day and the first day/post measurements (*p* = 0.03). On the 10th day, the mean d-ROMs value was still higher than both the first day values, but no significant differences were found.

All the BAP post-operative changes (Table 2) were not statistically significant compared to the first day/pre measurement. More specifically, the mean BAP value was increased immediately after mastectomy (first day/post) but decreased on the second post-operative day (when there was a significant increase in d-ROMs). Subsequently, the mean BAP value was increased again on the 3rd post-operative day (when d-ROMs mean value was further significantly increased) and on the 10th day (when d-ROMs value was lower than that on day 3).

Regarding the mean OSI value (Table 3), although it was elevated immediately after mastectomy and on the second or even the third post-operative day, no statistically significant differences between measurement times were found.

## 4. Discussion

Surgical trauma is considered the main cause for ROS production during the immediate post-operative period [10,11]. More specifically, due to surgical trauma and during the acute inflammatory phase of wound healing, large amounts of inflammatory cells such as neutrophils and macrophages are recruited at the site of surgical injury. These cells are known to induce ROS production not only through phagocytic activity, but also through the release of proinflammatory soluble mediators such as cytokines, chemokines and growth factors. In cancer cases, these soluble mediators activate a family of enzymes in cancer cells, the so-called Nox [nikotinamide adenine dinucleotide phosphate (NADPH) oxidase] enzymes that produce ROS [9,10,11,13]. As known, ROS facilitate cellular processes that are necessary for the dissemination of remaining tumor cells post-operatively [9,10,11,13,17,23,43]. In our study, increased numbers of WBCs and NEUT were found in blood, on the second and third post-mastectomy day (Figure 1), when there was also a significant increase in d-ROMs (Table 1). Similarly, in two recent studies [7,8] on the effect of anesthesia and surgery on host immunity in dogs with mammary cancer undergoing mastectomy, significantly increased numbers of WBCs and NEUT were found on day 3 post-surgery, when suppression in cell-mediated immunity and particularly T-lymphocytes (CD3^+^) and some of their subpopulations was also detected. It has been reported that in cancer cases, surgery-induced immunosuppression usually reaches a peak about day 3 post-surgery and lasts for about 10 days [3,5,7,8]. Therefore, although inflammatory cells are essential for surgical wound healing, they could also be involved in cancer progression, in the immediate post-operative period [6].

Oxidative stress plays an important role in breast cancer development and progression in both humans and dogs, as revealed from studies where increased levels of oxidation products and reduced antioxidants were found in the blood of cancer patients compared to healthy controls [17,24,26,27,42]. However, in a few other studies, various contradictory results have been reported [23,29,44].

Since many factors contribute to oxidative stress induction and a variety of antioxidants of endogenous (enzymatic and non-enzymatic) and exogenous origin are implicated [16,17], relevant studies have evaluated many different biomarkers of oxidant and particularly antioxidant status [17,18,23,26,29]. In the present study, the d-ROMs and BAP tests used for evaluating blood oxidant/antioxidant profile are considered to be two simple but accurate, reliable and suitable tools for clinical oxidative status evaluation in dogs [37,40]. Pasquini and colleagues [37], by using the same tests in a population of 130 healthy Labrador dogs aged 1–5 years, were the first that reported reference values for d-ROMs and BAP, which were from 54.4 to 91.4 CARR U and from 1440 to 3260 μmol/L, respectively.

In this study, the level of d-ROMs, which was pre-surgically in the upper limit of normal range according to the previously mentioned reference values [37], was significantly increased on the 2nd and 3rd post-operative day, but not on the 10th day (Table 1). Regarding the antioxidant profile, the mean BAP value changes after mastectomy (Table 2), although not statistically significant and always below the lower limit of normal range, might reflect efforts of the body to resist against the increased ROS production. Our findings, therefore, may reflect an inefficiency of host antioxidant defense system to compensate for the accumulation of ROS after mastectomy.

In a human study [26], in 35 breast cancer patients that were subjected to modified radical mastectomy, the pre-surgically elevated (compared to healthy controls) serum levels of the oxidative stress biomarker malondialdehyde (MDA) were found to be significantly decreased two weeks after surgery. In the same study, any changes found in serum levels of total antioxidant capacity (TAC) were not significant, as in our study. In a large number of dogs with malignant mammary tumors that were subjected to total (unilateral/bilateral) or regional mastectomy, a significant reduction in serum MDA levels was found 30 days after surgery compared to prior therapy values [24]. In dogs with mammary gland tumors subjected to mastectomy and ovariohysterectomy, the serum concentration of thiobarbituric acid reactive substances (TBARS), a marker of oxidative status, was found to be higher 10 min after surgery compared to 10 days after surgery [21]. In a study performed in human breast cancer patients, the high plasma total oxidant status (TOS) and OSI values and the low total antioxidant status (TAS) value compared to healthy controls (assessed 24 h before surgery) were found unchanged in the first post-operative month [42]. Generally, it would be expected that, in the early post-operative period, biomarkers of oxidant status would be found to be increased due to surgery and antioxidant ones being decreased, while in later periods, the opposite should be the case. However, according to the literature, as in the aforementioned studies, this is not always the case.

In experimental and clinical studies, it has been reported that some anesthetic or analgesic drugs can influence inflammation, immune responses, cancer cell proliferation and metastasis [3,43]. The results of studies on volatile anesthetics and opioids, such as isoflurane and morphine/fentanyl used in our study, are controversial [3,15]. It has been reported that isoflurane induced oxidative stress in human patients undergoing major surgeries, but it was considered safe in cases of minor surgeries [15]. On the other hand, propofol (used for induction of anesthesia in the present study) has shown anti-inflammatory and antioxidant properties, probably due to chemical similarity with other known antioxidants such as a-tocopherol [12,14,43]. In cancer surgery, therefore, the use of anesthetic drugs with antioxidant properties would be advisable. In the present study, it was decided to use an anesthetic/analgesic protocol that is commonly used in veterinary practice, although it included drugs that might increase oxidative stress. This decision was made in order to better reflect the situation in current veterinary clinical practice.

Although antioxidants play an important role in reducing oxidative stress, their role in preventing tumor growth and metastasis and the benefit of antioxidant supplementation in cancer treatment is controversial. Supplementation above physiological doses and taken for a long period of time could increase the risk of cancer or the survival/growth of cancer cells, and may be harmful in cancer chemotherapy and radiotherapy [11,18,20,21,28,30,31]. Nevertheless, the contribution of logical supplementation of antioxidant vitamins or other natural/synthetic antioxidant compounds to cancer prevention and treatment cannot be ignored [18,20].

Concerning the limitations of the present study, the wide weight range (7–28 Kg) and consequently the differences in body and wound size of the included animals could be considered as a limitation. However, the “extent of surgical wound”, a parameter calculated according to the body surface area, was considered as a safe inclusion criterion instead of wound size. Furthermore, our results on blood oxidant/antioxidant status could not have been influenced by parameters such as animal’s age or tumor type, since they were revealed from measurements over time (before and after surgery). However, the relatively small number of animals included in this study may have limited the possibility to detect any statistically significant differences in BAP, and subsequently, in OSI values between measurement times.

## 5. Conclusions

In bitches with mammary cancer, the significantly increased levels of d-ROMs on the second and third post-operative day may reflect an inability of the body to avoid the post-mastectomy accumulation of ROS and an oxidative stress increase. Further studies are needed to conclude whether interventions (e.g., use of anesthetic drugs with antioxidant properties or use of selective antioxidants for a short period peri-operatively) could be proved advisable for eliminating the post-surgical increase in oxidative stress, which might facilitate tumor cell dissemination and affect the patient’s outcome.

## Figures and Tables

**Table 1:** Measurements of d-ROMs (mean ± standard deviation) expressed in Carratelli Units (CARR U) in the blood serum of 12 bitches with mammary cancer undergoing mastectomy, on 1st day just before and after surgery (1st day/pre; 1st day/post) and on days 2nd, 3rd and 10th post-surgery.

Time	d-ROMs (CARR U); Mean ± Standard Deviation
1st day/pre	89.50 ± 18.9 ^a,b^
1st day/post	95.33 ± 23.6 ^c^
2nd day	105.08 ± 25.2 ^a^
3rd day	119.25 ± 20.3 ^b,c^
10th day	104.42 ± 25.4

Statistically significant differences between measurements, ^a^: *p* = 0.007; ^b^: *p* = 0.002); ^c^: *p* = 0.03; d-ROMs: Reactive Oxygen Metabolite-derived compounds.

**Table 2:** Measurements of BAP (mean ± standard deviation) expressed in μmol/L in the blood serum of 12 bitches with mammary cancer undergoing mastectomy, on 1st day just before and after surgery (1st day/pre; 1st day/post) and on days 2nd, 3rd and 10th post-surgery.

Time	BAP (μmol/L); Mean ± Standard Deviation
1st day/pre	1218.82 ± 605.1
1st day/post	1406.63 ± 725.9
2nd day	1070.46 ± 577.2
3rd day	1214.73 ± 631.7
10th day	1299.12 ± 654.5

BAP: biological antioxidant potential.

**Table 3:** Oxidative stress index (OSI) values (mean ± standard deviation) expressed in Arbitrary Units (AU) in the blood serum of 12 bitches with mammary cancer undergoing mastectomy, on 1st day just before and after surgery (1st day/pre; 1st day/post) and on days 2nd, 3rd and 10th post-surgery.

Time	OSI Value (AU); Mean ± Standard Deviation
1st day/pre	107.39 ± 94.8
1st day/post	175.14 ± 355.6
2nd day	198.18 ± 332.2
3rd day	117.27 ± 49.7
10th day	99.99 ± 57.3

OSI: the ratio of d-ROMs (in CARR U) and BAP (in mmol/L) values.

## Data Availability

The raw data supporting the conclusion of this article will be made available by the authors on request.
